# BSGE/ESGE guideline on management of fluid distension media in operative hysteroscopy

**DOI:** 10.1007/s10397-016-0983-z

**Published:** 2016-10-06

**Authors:** Sameer Umranikar, T. Justin Clark, Ertan Saridogan, Dimitrios Miligkos, Kirana Arambage, Emma Torbe, Rudi Campo, Attilio Di Spiezio Sardo, Vasilios Tanos, Grigoris Grimbizis, British Society for Gynaecological Endoscopy /European Society for Gynaecological Endoscopy Guideline Development Group for Management of Fluid Distension Media in Operative Hysteroscopy

**Affiliations:** 1 Princess Anne Hospital, Southampton, UK; 2 Birmingham Women’s Hospital, Birmingham, UK; 3 University College London Hospital, London, UK; 4 John Radcliffe Hospital, Oxford, UK; 5 St Michael’s Hospital, Bristol, UK; 6 Life Leuven, Leuven, Belgium; 7 University “Federico II” of Naples, Naples, Italy; 8 St’ Georges Med School, Nicosia University and Aretaeio Hospital, Nicosia, Cyprus; 9 Aristotle University of Thessaloniki, Thessaloniki, Greece

**Keywords:** Hysteroscopy, Hysteroscopic myomectomy, Endometrial resection, Distension medium, Fluid overload, Fluid pumps, Electrolyte imbalance


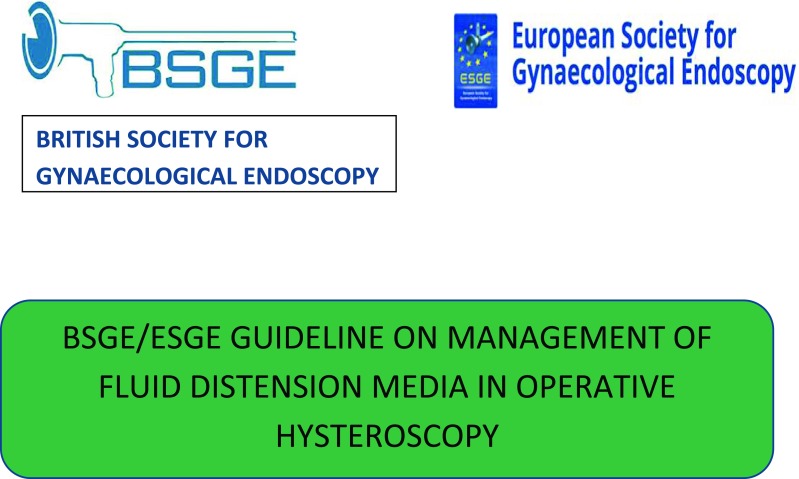


## Purpose and scope

The aim of this guideline is to provide clinicians with up-to-date, evidence-based information regarding management of distension media in operative hysteroscopy, with particular reference to prevention and management of complications that may arise from fluid overload.

## Identification and assessment of evidence

This guideline was developed using the methodology described by the RCOG for developing RCOG Green-top Guidelines (Clinical Governance Advice No.1: Development of RCOG Green-Top Guidelines (available on the RCOG website at http://www.rcog.org.uk/womens-health/clinical-guidance/development-rcog-green-top-guidelines-policies-andprocesses). The classification of evidence levels and grade of recommendations are given in Appendix 1.

The Cochrane Library (CENTRAL), MEDLINE (through PubMed), EMBASE (through Embase.com) were searched for potentially eligible records. We searched the databases using a combination of MeSH (Medical subject Headings) and relevant index terms. We used MeSH or index terms for the following key word: “operative hysteroscopy”, “TCRE”, “TCRF”, “hysteroscopy” AND “glycine”, “resectoscope”, “hysteroscopy” AND “fluid overload”, “hysteroscopy” AND “distension media”, “hysteroscopy” AND “management”. The search was limited to humans and papers in the English language. Relevant guidelines were also searched using the same criteria in the National Guidelines Clearinghouse, the National Electronic Library for Health, the Organising Medical Networked Information (OMNI) and the Canadian Medical Association (CMA) Infobase. The literature search, study selection and data extraction were carried out by two authors (SU and DM) independently, and a third author (JC) made the final decision in case of disagreement. Three authors (SU, DM and JC) graded the level of evidence.

## Introduction

Hysteroscopy enables visualisation of the uterine cavity and allows the diagnosis and surgical treatment of intrauterine pathology. To achieve this, the uterine cavity needs to be distended by a medium which could either be fluid or carbon dioxide [1]. Carbon dioxide is used for diagnostic hysteroscopy, as bleeding during operative procedures obscures visibility. For this reason, fluid media are used for operative procedures, as they allow continuous irrigation giving a clear picture and enable use of both mechanical and electrosurgical instruments. During operative hysteroscopy absorption of large volumes of distension solutions can occur leading to serious complications arising from significant fluid overload. Excessive fluid absorption is most likely with prolonged hysteroscopic procedures requiring continuous irrigation of fluid or where blood vessels within the myometrium are opened. Thus, particular care is required with resection of the endometrium (transcervical resection of the endometrium – TCRE) and hysteroscopic myomectomy – transcervical resection of fibroids – TCRF).

Operative hysteroscopic procedures are usually carried out using resectoscopes which are larger diameter, continuous flow operating hysteroscopes. They incorporate a working element that moves an electrically activated wire loop. These devices were initially developed to use monopolar current, which require non-electrolyte distending media such as glycine and sorbitol. Such solutions are however hypotonic so that excessive absorption can cause a number of complications including hyponatremia, a variable degree of hypo-osmolality, and certain solution-specific problems that are described below. Isotonic electrolyte-containing solutions cannot be used with monopolar energy because this leads to activation of ions that disperse the electric current and reduce the power density. Hence the heat generated in tissues is insufficient to destroy or have a tissue effect [2].

Resectoscopes have now been developed to use bipolar electrical current with the advantage that they are compatible with electrolyte-containing distension solutions such as physiological normal saline and Ringer’s lactate. Use of these solutions reduces the risk of hyponatremia, but excessive absorption can, as with monopolar current, lead to expansion of the extracellular fluid volume with the potential to generate fluid overload, pulmonary oedema, hypertension and cardiac failure.

Operative hysteroscopy can also be performed using small diameter, continuous flow hysteroscopes which incorporate a small, usually 5Fr or 7Fr, diameter working channel down which mechanical or electrosurgical instruments can be passed. Tissue removal systems refer to operative hysteroscopes that have been designed to simultaneously cut and aspirate tissue from within the uterine cavity. These systems usually incorporate their own fluid monitoring equipment but fluid overload can still occur. Smaller diameter operative hysteroscopes are less likely to cause fluid overload due to smaller diameter inflow channels and the generally less invasive nature of procedures that can be undertaken with such technology. However fluid overload may still occur and vigilance when using any operative hysteroscopic technology is mandatory.

## Fluid overload

### What is the definition of fluid overload?

**Table:** 

***A fluid deficit of more than 1000 ml should be used as threshold to define fluid overload when using hypotonic solutions in healthy women of reproductive age. [C]*** ***A fluid deficit of 2500 ml should be used as threshold to define fluid overload when using isotonic solutions in healthy women of reproductive age. [GPP]***	

Data evaluating fluid deficit during hysteroscopic surgery are lacking preventing a standard definition of fluid overload. A decrease in serum sodium of 10 mmol/L corresponds to an absorbed volume of approximately 1000 mL when using 1.5 % glycine [3] and it is for this reason that a fluid deficit of 1000 mL has traditionally been the threshold at which procedures should be curtailed in women of reproductive age when using hypotonic media.

With the advent of bipolar electrosurgical systems using isotonic solutions, fluid deficits >1000 mL will be tolerated by healthy women but a safe, upper limit is not still well defined and will depend upon an individual’s size, age and medical fitness.

In the absence of evidence to define an upper safe threshold for isotonic media the BSGE/ESGE Guideline Development Group recommends a limit of 2500 ml. This is in line with other national guidelines [4].

These thresholds apply to otherwise healthy fit women. However, in the elderly or those women with co-morbid conditions such as cardiovascular disease and renal impairment, lower thresholds should apply and it is suggested that upper fluid deficit levels of 750 ml for hypotonic solutions and 1500 ml for isotonic solutions [4].

### What should be the incidence of fluid overload during hysteroscopic surgery?

**Table:** 

***The incidence of fluid overload will vary according to case mix and type of hysteroscopic surgery. In general when using large diameter resectoscopes the incidence of fluid overloads should be less than 5 %. [D]*** ***The clinical course and outcome of all women with fluid overload should be audited. This should include unrecognised fluid overload in women presenting post-operatively as well as all women where fluid overload was identified during surgery. [GPP]***	

The incidence of fluid overload during hysteroscopic surgery is generally low [5]. Several prospective and retrospective studies have looked at the incidence of excessive fluid absorption and electrolyte disturbances during operative hysteroscopy and most report rates under 5 % [6–12]. Given the absence of a uniform definition of fluid overload, women manifesting with signs or symptoms of fluid overload post-hysteroscopic surgery should be recorded and followed up in the same way as those symptomatic and asymptomatic women who reach the pre-defined threshold for fluid overload.

## Complications of distending media

The complications of distension media during hysteroscopic surgery depend primarily on the type of medium used and the complexity of the operation. The main complications that occur are related to fluid absorption during the procedure leading to fluid overload with or without electrolyte imbalance.

### What factors pre-dispose to systemic fluid absorption?

**Table:** 

***Surgeons should understand the factors that can lead to systemic fluid absorption. High intrauterine distension pressure, low mean arterial pressure, deep myometrial penetration, prolonged surgery and large uterine cavities increase the likelihood of systemic fluid absorption. [GPP]***	

Absorption of distension media into the systemic circulation occurs by (i) retrograde passage of the fluid through the fallopian tubes, (ii) through the endometrium and (iii) via opened blood vessels and sinuses during resection of uterine tissue when the intrauterine pressure is greater than the pressure in the venous sinus or blood vessel. Factors influencing absorption of distension fluid include:

- *Intrauterine pressure* – the higher the pressure, the greater the degree of absorption into the body; systemic absorption of fluid increases considerably when intrauterine pressure exceeds mean arterial pressure [13]. In addition, intrauterine pressures > 75 mm Hg increases the volume of media passing back along the fallopian tubes and into the peritoneal cavity [14].
- *Mean arterial pressure* – the lower the mean arterial pressure, the lower the intrauterine pressure required to cause passage of fluid into the systemic circulation. Caution is thus required in the elderly and those with cardiovascular co-morbidities [15].
- *Depth of myometrial penetration* - when tissue damage extends into the deeper myometrium, instilled fluid can be rapidly absorbed through opened myometrial venous sinuses. The risk of fluid absorption is even greater during myomectomies where large blood vessels are breached facilitating the absorption of fluid under pressure.
- *Duration of surgery* – the longer the procedure the more time for fluid to accumulate within the body [16].
- *Size of uterine cavity* – larger cavities provide a greater endometrial surface area for fluid absorption and procedures will generally be longer. However, despite requiring more instilled fluid, high intrauterine pressures to allow adequate visualisation are harder to achieve [2].

Thus, rapid systemic fluid absorption is greatest with prolonged hysteroscopic procedures using large diameter endoscopes with high rates of media inflow creating significantly elevated intrauterine pressure and where uterine trauma and vessel transection occur such as with hysteroscopic myomectomy especially FIGO type I/II fibroids [17] metroplasty and endometrial resection.

### What factors influence the severity and nature of complications arising from excessive systemic fluid absorption?

**Table:** 

***Surgeons should understand the factors that influence the severity and nature of complications arising from excessive systemic fluid absorption. Severe complications are more likely with hypotonic (low osmolality) electrolyte free solutions, women of pre-menopausal status and those with cardiovascular or renal disease. [GPP]***	

Factors impacting on the propensity to serious complications arising from fluid overload include:

- *Osmolality of distension fluid* - hypotonic electrolyte-free solutions like glycine, mannitol and sorbitol can cause hyponatraemic hypervolaemia. If unrecognised and left untreated, bradycardia and hypertension can develop, rapidly followed by pulmonary oedema, cardiovascular collapse and death [18].
- *Menopausal status* - premenopausal patients have a higher risk of developing neurological complications due to the suppressive effects of oestrogen on the ATPase pump which regulates the flow of electrolytes through the blood brain barrier [19, 20].

*Cardiovascular and renal disease* – those women with known cardiovascular disease, renal impairment and the elderly are less likely to adapt to sudden significant increases in intravascular fluid such that complications from systemic fluid expansion and electrolyte imbalance are more likely at lower levels of fluid deficit [11].

### What complications arise from excessive systemic absorption of fluid distension media?

**Table:** 

***Surgeons should be aware of the potential complications when using different distension media during hysteroscopic surgery. These include morbidity and mortality arising from cardiovascular complications (pulmonary oedema and heart failure) and neurological complications (cerebral oedema, neurological impairment and seizures). [GPP]***	

The degree of systemic fluid absorption indicated by the size of the recorded fluid deficit and the type of the distension media will influence the presenting symptoms, type and severity of complications.

All types of fluid media can potentially cause complications where there is rapid systemic absorption, expansion of the systemic circulation leading to pulmonary oedema and heart failure. However, clinically significant fluid and electrolyte disturbances are more likely with hypotonic and electrolyte-free distension media [3] because they create an osmotic imbalance between extracellular and intracellular fluid. Conditions of hypo-osmolality and hyponatraemia cause water to move into brain cells inducing cerebral oedema, neurological impairment, seizures and even death [3]. Physiological isotonic solutions such as normal saline are less likely to cause such electrolyte disturbance [21].

### How do complications from excessive systemic absorption of fluid distension media present and how should they be managed?

**Table:** 

***Surgeons should be cognisant of cardiovascular and neurological symptoms associated with systemic absorption of fluid distension media complications to allow timely recognition and treatment. [D]*** ***Where excessive systemic absorption of fluid distension media is suspected, strict fluid balance monitoring should be commenced, a urinary catheter inserted and serum electrolytes measured. If the patient develops signs of cardiac failure or pulmonary oedema a cardiac echocardiogram and chest X-ray should be undertaken. [GPP]*** ***Asymptomatic hypervolemia with or without hyponatraemia should be managed by fluid restriction with or without diuretics. [GPP]*** ***The management of symptomatic hypervolemic hyponatraemia requires multidisciplinary involvement including anaesthetists, physicians and intensivists in a high dependency or intensive care unit. Initial treatment with 3 % hypertonic sodium chloride infusion is indicated to restore serum sodium concentrations to safe levels. [GPP]***	

#### Fluid overload with hypotonic fluid media

Glycine 1.5 % (200 mOsm/L) and sorbitol 3 % (165 mOsm/L) are the most common hypotonic electrolyte-free distending media used for operative hysteroscopy with monopolar electrosurgical energy. Moderate fluid overload causes hypervolaemia and consequent dilutional hyponatraemia. At that stage, despite the drop of sodium concentration, the osmolality of the blood is not greatly affected (normal osmolality, 280 mOsm/L). This asymptomatic hyponatraemia can be managed with fluid restriction and diuretics such as frusemide in the absence of a diuresis.

Symptoms usually develop when serum sodium concentration drops below 125 mmol/L. The most frequent symptoms are headache, nausea, vomiting and weakness. If further fluid intravasation occurs, reduction of the blood osmolality creates an osmotic gradient that moves water into the interstitial and intracellular space, leading to brain oedema and increased intracranial pressure. The resultant cerebral oedema may present with signs of cerebral irritation such as agitation, apprehension, confusion, weakness, nausea, vomiting, visual disturbances, blindness and headache. If significant, it can lead to brain stem herniation, coma and death [22]. A further fall of sodium below 120 mmol/L may lead to confusion, lethargy, seizures, coma, arrhythmias, bradycardia and respiratory arrest.

Thus, early recognition and treatment is essential to prevent cardiovascular complications and permanent neurological sequelae resulting from toxic hyponatraemia. A strict fluid balance must be commenced in theatre and should extend into the postoperative period. A urinary catheter should be inserted and the electrolytes, urea and creatinine measured. A loop diuretic like frusemide should be given intravenously and the urine output measured. If the patient develops signs of cardiac failure or pulmonary oedema a cardiac echocardiogram and chest X-ray should be undertaken with involvement of the physicians.

The management of symptomatic hyponatraemia requires multidisciplinary involvement including anaesthetists, physicians and intensivists in a high dependency or intensive care unit (Table 1). Intravenous infusion of a slow 3 % hypertonic sodium chloride infusion (typically 1–2 mmol/L/h to prevent pontine myelinolysis) is indicated until serum sodium rises to 125 mmol/ [2, 18, 22, 23] correcting any cerebral oedema and reducing the risk of systemic complications. Acute hyponatraemia below 120 mm/l and/or acute symptomatic hyponatraemia should be treated with a 100 ml bolus of 3 % saline over 10 min and this can be repeated up to three times, followed by an infusion as described above. The recommended target increase of the serum sodium is 6 mmol/L over 24 h until 130 mmol/L is reached. Even a small increase in the sodium concentration can reduce the risk of cerebral oedema and its [24, 25]. The clinical condition and observations such as oxygen saturations, urine output and serum electrolytes including potassium and calcium should be closely monitored.

**Table 1:** Management of suspected hypervolaemic hyponatraemia arising from fluid overload >1000 ml with hypotonic distension media

Acute hypervolaemic hyponatraemia^a^	Management
Asymptomatic hyponatraemia & [Na^+^] ≥120 mmol/L	Fluid restriction (e.g., <1 L/day) and loop diuretics e.g.,40 mg frusemide
Symptomatic hyponatraemia and/or [Na^+^] <120 mmol/L	Hypertonic (3 %,) saline (1 L = 513 mmol/L NaCl compared with normal saline where 1 L = 154 mmol/L), supplemental oxygen, indwelling urinary catheter, high dependency care and multidisciplinary team involvement

^a^normal serum sodium levels are approximately between 135 and 145 mmol/L

Sorbitol 3 % is a hypotonic sugar solution and if excessive intravasation of sorbitol occurs, it can also lead to hyperglycaemia and hypocalcemia. Consequent symptoms can develop quite rapidly, as myoclonus within an hour of the procedure has been described [26, 27]. In this situation, monitoring of the blood sugars is necessary and starting an insulin sliding scale if the blood sugar levels are high. Hypocalcemia should be corrected with 3 g of calcium gluconate over 10 min [28]. This should be given with advice from an intensivist.

#### Fluid overload with isotonic fluid media

Bipolar electrosurgery is conducted in electrolyte containing solutions such as physiological saline. This medium reduces the risk of hypo-osmolarity and hyponatremia with excessive fluid absorption but does not eliminate the risk of congestive cardiac failure and pulmonary oedema. Fluid restriction, diuretics and monitoring as described above is usually all that is required.

### What volume of fluid absorption is required to cause significant hyponatraemia and hypervolaemia?

**Table:** 

***Fluid absorption of over 1000 ml of hypotonic solution can cause clinical hyponatraemia. [D]*** ***Mild symptoms can develop even with absorption of 500–1000 ml of a hypotonic solution. [C]*** ***Larger volumes of isotonic solution need to be absorbed to cause symptomatic fluid overload but there are no data to define a safe threshold. [D]***	

In a study by Magos et al. [29] with glycine intravasation of 1000 ml, the plasma sodium fell by 7–8 mmol/L and the authors concluded that this fall in sodium was sufficient to cause hyponatraemia to a patient with a previously normal serum sodium concentration. A further study by Istre et al. [3] demonstrated a significant drop in the serum sodium concentration of up to 10 mmol/L when more than 1000 ml of electrolyte free hypotonic fluid was absorbed during surgery. An extreme drop in serum sodium concentration to 83 mmol/L has been reported with the use of a combined sorbitol / mannitol solution [30]. The first signs of hyponatraemia can present with fluid deficits of 500 ml. In a small prospective study, 20 patients underwent a transcervical resection of the endometrium (TCRE) of which 10 patients with postoperative nausea demonstrated glycine absorption of more than 500 ml whereas none of the patients with deficit of less than 500 mL complained of nausea. Eight patients who had glycine absorption of more than 1000 ml showed evidence of cerebral oedema on CT scan. The authors concluded that cerebral oedema may contribute to the development of postoperative nausea in patients undergoing operative hysteroscopy and who absorb more than 500 ml of 1.5 % glycine [3].

Normal saline is an isotonic solution and therefore excessive fluid absorption is not associated with electrolyte disturbances. However it can result in hypervolaemia, pulmonary oedema and congestive heart failure. There is a risk of tissue oedema which can lead to poor tissue oxygenation and altered pulmonary gas function [31]. Usually these complications can be reversed with administration of diuretics. Isotonic fluids like sodium chloride used with bipolar resectoscopes may reduce complications related to electrolyte disturbances however similar principles of fluid management should be applied to all cases and the procedure terminated if excess fluid has been absorbed.

### Which is the safest distension medium to avoid complications from fluid overload?

**Table:** 

***Isotonic media are safer than hypotonic media as fluid absorption does not cause hyponatraemia. [A]*** ***Fluid deficit should still be closely monitored when using either hypotonic or isotonic distension media. [GPP]***	

In addition to avoiding excess fluid absorption, prompt recognition of fluid overload and instigating appropriate treatment is important. The prevention of complications from distending media requires the selection of the solution least likely to cause serious complications should it be absorbed in excess.

The use of isotonic fluid in operative hysteroscopy is considered safer as fluid absorption does not cause hyponatraemia. However the exact volume of normal saline absorption that is safe is not known. In theory this could be higher than hypotonic solutions [32]. In a randomised study of 200 pre-menopausal women undergoing operative hysteroscopy with monopolar versus bipolar energy using glycine 1.5 % and normal saline respectively, the authors documented a statistically significant reduction of sodium from 138.7 to 133.8 mmol/L when glycine was used whereas there was no change in the sodium concentration in the saline group [33]. However they found the fluid deficit to be significantly higher in the normal saline group. They concluded that resectoscopes using bipolar energy have a safer profile than monopolar energy due to the unchanged sodium concentrations. The increased fluid deficit was not accompanied by any complications during or after the procedure. Similar findings were also noted in a prospective randomized study where 155 women undergoing hysteroscopic myomectomy were randomized to 0.9 % saline versus 1.5 % Glycine. The authors found no change in the sodium levels or the osmolality in the women who underwent treatment with normal saline [34].

However even with normal saline, close vigilance to the fluid deficit is imperative as there are case series where large volumes of fluid have been absorbed leading to excessive fluid overload and pulmonary oedema [35, 36].

### What amount of fluid overload should be considered safe when undertaking hysteroscopic surgery?

**Table:** 

***A maximum fluid deficit of 1000 ml should be set when using a hypotonic solution in a healthy woman and surgery immediately stopped on reaching this limit [C]*** ***A maximum fluid deficit of 2500 ml should be set when using an isotonic solution in a healthy woman and surgery immediately stopped on reaching this limit [GPP]*** ***Lower thresholds for fluid deficit should be considered in the elderly and women with cardiovascular, renal or other co-morbidities. Suggested upper limits are 750 ml for hypotonic solutions and 1500 ml for isotonic solutions although these limits may need to be reduced depending upon the clinical condition of the woman during surgery. [GPP]*** ***The fluid deficit threshold should be agreed with the anaesthetist pre-operatively and the overall fluid deficit and estimated intravascular component should be communicated to the anaesthetist at the end of the procedure to guide post-operative care. [GPP]***	

When a deficit of isotonic solution such as normal saline reaches 1500 mL or a deficit of hypotonic solution reaches 750 mL, the surgeon should inform the anaesthetist and the nursing team and monitor the subsequent surgical period with special care. Detailed evaluations have to be performed and documented before decision to finalise the surgery can be made. The Guideline Development Group’s consensus view is that once a fluid deficit of 1000 ml of hypotonic solution or 2500 ml with an isotonic solution is reached immediate suspension of the procedure is imperative.

These thresholds apply to otherwise healthy fit women. However, in the elderly or those women with co-morbid conditions such as cardiovascular disease and renal impairment, lower thresholds should be applied (i.e., 750 ml for hypotonic solutions and 1500 ml for isotonic solutions). In these latter groups of patients, the threshold for fluid loss should be agreed in advance of surgery between the surgeon and anaesthetist and procedures curtailed sooner if signs of fluid overload and / or hyponatraemia become apparent.

When the relevant fluid deficit threshold has been reached and the procedure stopped, an attempt at estimating the intravascular component of the fluid loss can be made. Fluid collected in drapes, in urine and passed via the fallopian tubes into the peritoneal cavity is less clinically concerning than that infused directly into intravascular system. Thus, drapes can be visualised and even weighed to estimate fluid content, urine output measured by catheterising the bladder and ultrasound used to estimate the amount of fluid in the peritoneal cavity. In this way an estimate of the total intravascular fluid administration can be made. Communication with the anaesthetist is important to guide post-operative fluid management as they will also be aware of the amount of intravenous fluid given peri-operatively.

### Can air or gas embolism occur during a hysteroscopic procedure?

**Table:** 

***Clinically significant gas or air embolism is a rare complication of hysteroscopy. However this diagnosis should be considered if the patient develops sudden oxygen desaturation or cardiovascular collapse during the procedure. [D]***	

Air or gas embolism is rare but can occur during a hysteroscopy with both gas (CO_2_) or fluid distension media and in the outpatient as well as inpatient setting [37, 38]. Air can enter the uterine cavity during insertion of the hysteroscope if the inflow tubing is not primed with fluid or due to air bubbles within the distension medium potentially causing air embolism [39]. To minimise the risk of air embolism, the hysteroscope and inflow tubing should be primed with the fluid media to eliminate air bubbles before inserting the hysteroscope into the uterine cavity [37]. Gas embolism may arise from the combustion of gases produced during hysteroscopic electrosurgery [40]. The gases normally produced are primarily carbon dioxide (CO_2_) as well as carbon monoxide and evaporative gases, the latter being easily soluble in blood and hence do not cause serious complications [40].

In a randomized study [41] of 50 patients, venous gas embolism was seen in all but one patient when using transoesophageal echocardiography. They found the incidence to be higher with bipolar energy and when the fluid absorbed was more than 1000 ml. There have been reported cases of mortality due to life threatening complications using dextran under high intrauterine pressure [42]. Clinically significant gas embolism is considered to be quite rare and there are relatively few case reports published in the literature [35, 43]. If an embolism occurs, the vital signs and oxygen saturation of the patient can acutely deteriorate with subsequent cardiovascular collapse [44]. The pathophysiology involved in an embolic phenomenon is the passage of the embolus to the pulmonary circulation, initially creating a high ventilation/ perfusion ratio which reduces the end tidal carbon dioxide. This causes diversion and overperfusion of the pulmonary blood flow to the unaffected areas within the lung, away from the embolised area leading to a low ventilation/ perfusion ratio causing hypoxaemia [40]. In addition the physical presence of the embolus can cause mechanical obstruction which could lead to cardiac strain and cardiovascular collapse. In this situation the patient needs to be resuscitated and managed in an intensive care setting.

With CO_2_ embolism, the frequency of severe adverse events is rare due to the properties of CO_2_ gas which is soluble in the blood and gets readily eliminated from the respiratory system. However use of inappropriate equipment such as a laparoscopic insufflator to instill CO_2_ during hysteroscopic surgery, has been acknowledged to cause these complications and the surgeons should ensure that the correct equipment is used [45].

Thus clinically significant complications associated with gas or air embolism are rare but the surgeon and anaesthetist should be aware of these potentially life threatening complications.

## Choosing distension media

### What type of distension media should be used for operative hysteroscopy?

**Table:** 

***Isotonic electrolyte-containing distension media such as normal saline should be used with mechanical instrumentation and bipolar electrosurgery because they are less likely to cause hyponatraemia if fluid overload occurs. [D]*** ***Hypotonic, electrolyte-free distension media such as glycine and sorbitol should only be used with monopolar electrosurgical instruments. [D]*** ***Carbon dioxide gaseous media should not be used for operative hysteroscopy. [GPP]***	

Fluid media are most suitable when undertaking operative procedures. The advantage of fluid over CO_2_ gas is the symmetric distension of the uterus with fluid and its effective ability to flush blood, mucus, bubbles, and small tissue fragments out of the visual field [1]. Presence of blood and debris and the risk of gas embolism render CO_2_ unsuitable to use in operative hysteroscopy. Amongst the fluid media, the choices are between an isotonic or hypotonic fluid depending upon the energy modality used. Isotonic fluids may contain electrolytes such as sodium chloride and Ringer’s lactate solution or are electrolyte free such as mannitol, although the latter is rarely used in the UK. Electrolyte solutions are used with bipolar energy and with mechanical procedures such as morcellation of submucosal fibroids and endometrial polyps. Electrolyte containing fluids would not be effective when using monopolar energy, as energy would get dissipated during the surgical procedure; hence electrolyte free solutions have to be used with monopolar energy. The characteristics and osmolality of the different fluid media are further described in Table 2.

**Table 2:** Types of distension media and their applicability in operative hysteroscopy

Distension Media [Normal Plasma osmolality (285 mOsm/L)]	Procedure	Electrolyte free	Osmolality	Energy	Comments
Normal saline 285 mOsm/L Ringer’s lactate 279 mOsm/L	Diagnostic and Operative hysteroscopy Diagnostic and Operative hysteroscopy	No No	Iso-osmolar	Mechanical Bipolar Laser	Not recommended with monopolar energy as it disperses electric current without having any surgical effect on the tissue
Glycine 1.5 % 200 mOsm/L	Operative Hysteroscopy	Yes	Hypo-osmolar	Monopolar	
Dextrose 5 %	Operative Hysteroscopy	Yes	Hypo-osmolar	Monopolar	
Sorbitol 3 % 165 mOsm/L	Operative Hysteroscopy	Yes	Hypo-osmolar	Monopolar	
Mannitol 5 % 274 mOsm/L	Operative Hysteroscopy	Yes	Iso-osmolar	Monopolar	

The high viscosity fluid dextran, and the isotonic, electrolyte-free low viscosity fluid mannitol are rarely used in the UK and Europe

The solutions available can be either of high or of low viscosity. The high viscous solution like Dextran 32 % produces good visualization of the cavity in the presence of blood as it is immiscible with blood. However it is known to cause anaphylactic reactions [46] and can also lead to crystallization within the telescope rendering it to damage if not cleaned properly immediately after the procedure. Furthermore, it is hyperosmolar and even small volumes absorbed can lead to disproportionate intravascular expansion and cardiac failure. Low viscosity fluids can be divided into isotonic or hypotonic in relation to the osmolality of plasma which is around 285 mOsm/L. Low viscosity fluids which are isotonic are 0.9 % normal saline, Ringer’s lactate and 5 % mannitol. Low viscosity fluids which are hypotonic are 1.5 % Glycine, 3 % sorbitol and 5 % dextrose (Table 2).

The ideal distending medium should allow clear visualisation of the uterine cavity, be isotonic, nontoxic, hypoallergenic, non-haemolytic, be rapidly cleared by the body, readily available and inexpensive. With the advent of bipolar electrosurgery, a conductive electrolyte containing medium is advantageous. Normal saline satisfies all these criteria and for this reason appears to be the fluid distension medium of choice for mechanical hysteroscopic surgery and bipolar electrosurgery.

## Strategies to reduce fluid absorption

### What preoperative measures can be taken to reduce fluid absorption?

**Table:** 

***Preoperative administration of GnRH agonists should be considered in premenopausal women before hysteroscopic resection of fibroids. [B]*** ***Intracervical injection of dilute Vasopressin can be considered before dilatation of the cervix. [B]***	

Gonadotrophin releasing hormone (GnRH) agonists induce amenorrhoea, improve anaemia, prepare the endometrium prior to hysteroscopic interventions and can reduce the volumes of submucosal fibroids enabling TCRF. In addition, GnRH agonists can reduce electrolyte disturbance complications in premenopausal women by enhancing the action of the sodium-potassium ATP-ase pump responsible for shunting sodium outside the cells. This pump is inhibited by female sex steroids making premenopausal women more susceptible to hyponatraemic complications during hysteroscopic surgery [20, 21].

Studies have shown that giving pre-operative GnRH analogues when undertaking resection of the myoma or endometrium reduces the incidence of fluid overload [47, 48].

A small RCT by Taskin et al. [19] showed statistical difference in the reduction of serum sodium concentration but not in glycine deficit in women treated preoperatively with GnRH agonists. Another RCT [49] did not show a statistically significant difference in fluid deficit with the use of GnRH agonists.

Three studies have shown that intracervical injection of diluted Vasopressin immediately before cervical dilatation is associated with reduced fluid absorption during operative hysteroscopy [50–52]. Extreme caution and communication with the anaesthetic team should be undertaken as systemic absorption of large doses of vasopressin can lead to cardiovascular collapse and death.

### What intraoperative measures can be taken to reduce fluid absorption?

**Table:** 

***The intrauterine pressure needed for distension should be maintained as low as possible to allow adequate visualisation and kept below the mean arterial pressure. [B]***	

For visualisation of the uterine cavity, a distension medium is required to separate the uterine surfaces, needing an intrauterine pressure (IUP) of between 70 and 100 mmHg. The pressure needed depends on the uterine size, muscle thickness and tone. The higher the IUP is, the higher the risk of excessive fluid absorption. The pressure within the venous sinuses in the myometrium is thought to be around 10–15 mmHg [53]. Once the IUP exceeds the mean arterial pressure (normal 70 to 110 mmHg), then significant amount of fluid can get absorbed in the circulation [13]. In a small randomized study [14] 26 women were randomized to either having uncontrolled IUP (mean maximum 135 mmHg) or controlled IUP (mean maximum of 70 mmHg) during surgery. They found a fluid deficit of 1255 ml in the uncontrolled group as compared to no fluid deficit in the controlled group of women highlighting the importance of avoiding high IUP during surgery. Control of the IUP has been shown to reduce the amount of fluid absorption by almost 85 % [13]. Some studies have suggested keeping the IUP between 45 and 80 mmHg as pressures may exceed the mean arterial pressure increasing the likelihood of rapid fluid absorption [54, 55]. For short procedures a minimum intrauterine pressure of around 40 mmHg is feasible [56]. Filling pressures of up to 100 mmHg have been found to be effective and safe in outpatient hysteroscopy [57].

In a randomized study [58] 48 women undergoing TCRE for abnormal uterine bleeding, were randomised to intravenous oxytocin infusion or saline infusion intraoperatively. There was a significant lower glycine deficit and decrease in serum sodium concentration in the oxytocin group, however the numbers in their study were quite small.

## Delivery of distension media

### Methods used for delivering distension media

**Table:** 

***Delivery of the distension medium can be safely and effectively achieved using simple gravity, pressure bags or automated delivery systems. [D]*** ***Automated pressure delivery systems facilitate the creation of a constant intrauterine pressure and accurate fluid deficit surveillance which is advantageous with prolonged cases such as endometrial resection or hysteroscopic myomectomy. [D]***	

Intrauterine distension pressure can be maintained using simple gravity, manual and automated pressure delivery systems. Simple gravity systems deliver the distension fluid by hydrostatic pressure. The achieved pressure in the inflow port of the hysteroscope is the product of the width of the inflow tube and the level difference between the highest portion of the fluid column and level of the uterus. Elevation of the bag will increase the intrauterine pressure and one foot of height will approximate to around 25 mm of Hg [59]. When the fluid is maintained at a level of 1 to 1.5 m above the patient’s uterus, the intrauterine pressure will be between 70 to 100 mmHg [21].

Manual pressure systems maintain the necessary intrauterine pressure by using a pressure bag or blood pressure cuff around the fluid bag. The disadvantage of all these systems is that they keep the flow and the pressure at the inflow port constant and therefore if the pressure exceeds the mean arterial pressure it can lead to excessive fluid absorption. Irrigation of fluid is achieved by opening partially or fully the outflow tap and applying varying amounts of negative pressure (suction).

A variety of automated fluid pumps exist. Some will keep a constant pre-set pressure at the inflow port but they will continue delivering fluid despite the resistance in the uterine cavity. Other systems will titrate the intrauterine fluid pressure constantly at 70–80 mmHg and will reduce the fluid flow (inflow and outflow) accordingly. A device which maintains constant intrauterine pressure is more sensitive and limits excessive intrauterine pressures and subsequent intravasation of the distending medium [15]. These systems can be costly to set up and run. They are not necessary for short operative or diagnostic procedures but maybe beneficial for prolonged, operative cases such as resection of the endometrium or submucosal fibroids where endometrial and myometrial disruption occur causing bleeding and the formation of intrauterine tissue debris that can compromise visualisation of the operative field.

It is important to bear in mind that measurement and maintenance of the intrauterine pressure can be difficult when there is leakage of fluid around the cervix especially after use of cervical priming agents [60] and when suction is applied at the outflow port of the hysteroscope.

## Monitoring fluid deficit

### How should fluid deficit be measured during operative hysteroscopy?

**Table:** 

***Mechanisms should be in place to monitor fluid deficit during operative hysteroscopic surgery. [GPP]*** ***Closed systems should be used as they allow more accurate measurement of the fluid output. [GPP]*** ***Drapes that contain a fluid reservoir should be used as they allow measurement of the fluid output. [GPP]*** ***Automated fluid measurement systems are more accurate than manual measurement but they can still overestimate fluid deficit. Their use cannot guarantee safety but might be useful when undertaking complex hysteroscopic procedures where fluid absorption is anticipated. [D]***	

Inflow and outflow fluid monitoring involves the calculation of the volume infused in the uterine cavity and the fluid returned from the outflow channel of the hysteroscope and the fluid leaking through the cervix. Liquid media can be delivered into the uterine cavity via an open or closed system. In an open system the medium freely escapes through the cervix and the outflow channel onto the drape and into a bucket or the theatre floor, thus making precise fluid monitoring inaccurate or even impossible. In a closed system the fluid is returned through suction to a reservoir. This set up also improves visibility by removing debris and blood from the endometrial cavity. However, even with suction, there is still fluid escaping through the cervix and the perineum. To overcome this problem drapes with a fluid reservoir for collection of fluid should be used. The fluid collected in the reservoir can be measured and added in the outflow volume. These drapes should be used instead of the standard surgical drapes.

Measurement of fluid deficit should be undertaken by a dedicated member of the theatre team. Measurement may be subject to errors if the member of staff responsible has other duties as well. Each unit should designate a member of the theatre team to carry out the fluid measurement, calculate the fluid balance and communicate it to the surgeon. The accurate estimation of fluid used from bags can be problematic. A study of bags of normal saline, glycine, and sorbitol found that the average overfill was between 3 and 6 % of the bag volume therefore this fact should be taken into consideration [61] when fluid deficit is calculated. Also accurate estimation of fluid within the bags during and at the end of the procedure is poor and errors can range from 4 to 50 % [62]. Significant bleeding during hysteroscopic surgery can also make fluid deficit calculation less reliable, as the outflow may appear more than the actual value, giving a false lower deficit.

To overcome the limitations of manual measurement a variety of automated systems have been developed. These will continuously calculate the fluid deficit by measuring the weight difference between the inflow fluid bags and the reservoirs collecting the outflow fluid. Some are also designed to give an alarm if there is a suspected perforation and the fluid loss is more than 300 ml/min or give automated alerts at every 250 ml of fluid deficit. Automated systems also compensate for the discrepancy in the actual volume of fluid in the bags. The theatre team should be cautious to ensure there is no fluid escaping from the drapes, as this can overestimate the fluid deficit. The costs of automated systems have precluded widespread usage but they appear to be beneficial in prolonged cases where fluid absorption is anticipated e.g., TCRE, TCRF, adhesiolysis and septoplasty.

### How often should fluid deficit be calculated?

**Table:** 

***Measurement of the fluid deficit should be done at a minimum of 10 min intervals during hysteroscopic surgery. [GPP]***	

There is no evidence on the optimal monitoring frequency for estimating fluid deficit. The consensus of the guideline authors is that the theatre team should keep a running balance at least every 10 min and at the end of usage of each fluid bag. Sometimes in cases of uterine perforation the fluid bag will be consumed quite quickly and this should alert the surgeon and theatre team. The running fluid balance should be communicated with the operating surgeon and the anaesthetist and documented (see Appendix 2).

## Anaesthesia and impact upon fluid overload and electrolyte imbalance

**Table:** 

***Where feasible, the use of local anaesthesia with sedation should be considered for performing operative hysteroscopic procedures rather than general anaesthesia because fluid overload may be minimised [B]***	

In a RCT [63] comparing type of anaesthesia, women who underwent operative hysteroscopic procedures (endometrial resection with or without polypectomy or myomectomy) under general anaesthesia had a higher median glycine absorption compared to women who underwent these procedures using local paracervical anaesthesia with 1 % lidocaine and midazolam, sufentanil and propofol sedation (480 mL [76–1300 mL] versus 253 mL [70–728 mL], *p* = 0.01). General anaesthesia was also associated with a higher rate of glycine absorption >1000 mL (20 % versus 4 %), a greater fall in serum sodium concentration (2.0 meq/L versus 0.5 meq/L and a greater rate of fall in sodium concentration ≥10 meq/L (8 % versus 0 %) than local anaesthesia with sedation. However, cases deemed suitable for local anaesthesia and hence eligible for randomisation are likely to have been less complex, short duration procedures limiting the generalisability of these findings. Another small RCT with 24 patients compared general with epidural anaesthesia during endometrial resection. There was significantly lower glycine deficit in the general anaesthesia group [64].

## Suggested audit topics

- Proportion and type of hysteroscopic procedures exceeding recommended fluid deficits and exploration of clinical outcomes
- Prevalence of fluid distention media complications and compliance with guidance presented for subsequent management.
- Impact of innovations to reduce fluid overload on subsequent prevalence

## Recommendations for research

- Safe maximum fluid deficit thresholds during operative hysteroscopy for isotonic fluids
- Effectiveness of automated fluid delivery systems in reducing fluid distension media complications
- Effectiveness and safety, including fluid distention media complications, of new hysteroscopic tissue removal systems compared with conventional electrosurgery for operative hysteroscopic procedures such as myomectomy.

The distension media related complications are relatively uncommon and RCTs to study the safety of surgical procedures would need large numbers of study entrants. Data on safety and complications may be more adequately collected prospectively from multiple centres within a reasonable amount of time. Online registries under the auspices of large international societies such as the ESGE may be useful tools to measure the incidence of complications before and following the introduction of new techniques, instruments and clinical practice guidelines.

### Executive summary of recommendations

Good practice point - Recommended best practice based on the clinical experience of the guideline development group.

#### Incidence of fluid overload

- A fluid deficit of more than 1000 ml should be used threshold to define fluid overload when using hypotonic solutions in healthy women of reproductive age. **[C]**
- In the absence of a consensus, a fluid deficit of 2500 ml should be used threshold to define fluid overload when using isotonic solutions in healthy women of reproductive age. **[GPP]**
- The incidence of fluid overload will vary according to case mix and type of hysteroscopic surgery. In general when using large diameter resectoscopes the incidence of fluid overloads should be less than 5 %. **[D]**
- The clinical course and outcome of all women with fluid overload should be audited. This should include unrecognised fluid overload in women presenting post-operatively as well as all women where fluid overload was identified during surgery. **[GPP]**

#### Complications of distending media

- Surgeons should understand the factors that can lead to complication related to distension media. **[GPP]**
- Surgeons should understand the factors that influence the severity and nature of complications arising from excessive systemic fluid absorption. **[GPP]**
- Surgeons should be aware of the potential complications when using different distension media during hysteroscopic surgery. **[GPP]**
- Surgeons should be cognisant of symptoms associated with distension media complications to allow timely recognition. **[D]**
- Fluid absorption of over 1000 ml of hypotonic solution can cause clinical hyponatraemia. **[D]**
- Mild symptoms can develop even with absorption of over 500 ml of a hypotonic solution. **[C]**
- Larger volumes of isotonic solution need to be absorbed to cause symptomatic fluid overload but there are no data to define a safe threshold. **[D]**
- Isotonic medium is considered safer than hypotonic media as fluid absorption does not cause hyponatraemia. **[A]**
- Fluid deficit should still be closely monitored when using either hypotonic or isotonic distension media. **[GPP]**
- A maximum fluid deficit of 1000 ml should be set when using a hypotonic solution in a healthy woman and surgery immediately stopped on reaching this limit. **[C]**
- A maximum fluid deficit of 2500 ml should be set when using an isotonic solution in a health woman and surgery immediately stopped on reaching this limit. **[GPP]**
- Lower thresholds for fluid deficit should be considered in the elderly and women with cardiovascular, renal or other co-morbidities. Suggested upper limits are 750 ml for hypotonic solutions and 1500 ml for isotonic solutions although these limits may need to be reduced depending upon the clinical condition of the woman during surgery. **[GPP]**
- Most serious complications associated with fluid media are related to excessive absorption during surgery and rarely gas or air embolism during the procedure. **[D]**

##### Choosing distension medium

- Isotonic, electrolyte-containing distension media such as normal saline should be used with mechanical instrumentation and bipolar electrosurgery because it is less likely to cause hyponatraemia where there is fluid overload. **[D]**
- Hypotonic, electrolyte-free distension media such as glycine and sorbitol should only be used with monopolar electrosurgical instruments. **[D]**
- Carbon dioxide gaseous media should not be used for operative hysteroscopy. **[GPP]**

##### Strategies to reduce fluid absorption

- Preoperative administration of GnRH agonists should be considered in premenopausal women before hysteroscopic resection of fibroids. **[B]**
- Intracervical injection of dilute Vasopressin can be considered before dilatation of the cervix. **[B]**
- The intrauterine pressure needed for distension should be maintained as low as possible to allow adequate visualisation and kept below the mean arterial pressure. **[B]**

#### Delivery of distension media

- Delivery of the distension medium can be safely and effectively achieved using simple gravity, pressure bags or automated delivery systems. **[D]**
- Automated pressure delivery systems facilitate the creation of a constant intrauterine pressure and accurate fluid deficit surveillance which is advantageous with prolonged cases such as endometrial resection or hysteroscopic myomectomy. **[D]**

#### Monitoring fluid deficit

- Mechanisms should be in place to monitor fluid deficit during operative hysteroscopic surgery. **[GPP]**
- Closed systems should be used as they allow more accurate measurement of the fluid output. **[GPP]**
- Drapes that contain a fluid reservoir should be used as they allow measurement of the fluid output. **[GPP]**
- Automated fluid measurement systems are more accurate than manual measurement but they can still overestimate fluid deficit. Their use should be considered for prolonged complex hysteroscopic procedures where fluid absorption is anticipated. **[D]**
- Measurement of the fluid deficit should be done at a minimum of 10 min intervals during hysteroscopic surgery. **[GPP]**

#### Anaesthesia and impact upon fluid overload and electrolyte imbalance

- Where feasible, the use of local anaesthesia with sedation should be considered for performing operative hysteroscopic procedures rather than general anaesthesia because fluid overload may be minimised. **[B]**

## Appendix 1

### Classification of evidence levels

**1++** High-quality meta-analyses, systematic reviews of randomised controlled trials or randomised controlled trials with a very low risk of bias

**1+** Well-conducted meta-analyses, systematic reviews of randomised controlled trials or randomised controlled trials with a low risk of bias

**1–** Meta-analyses, systematic reviews of randomised controlled trials or randomised controlled trials with a high risk of bias

**2++** High-quality systematic reviews of case–control or cohort studies or high-quality case-control or cohort studies with a very low risk of confounding, bias or chance and a high probability that the relationship is causal

**2+** Well-conducted case-control or cohort studies with a low risk of confounding, bias or chance and a moderate probability that the relationship is causal

**2-** Case-control or cohort studies with a high risk of confounding, bias or chance and a significant risk that the relationship is not causal

**3** Non-analytical studies, e.g., case reports, case series

**4** Expert opinion

### Grades of recommendations

**A -** At least one meta-analysis, systematic review or randomised controlled trial rated as 1++ and directly applicable to the target population; or

A systematic review of randomised controlled trials or a body of evidence consisting principally of studies rated as 1+ directly applicable to the target population and demonstrating overall consistency of results

**B -** A body of evidence including studies rated as 2++ directly applicable to the target population, and demonstrating overall consistency of results; or

Extrapolated evidence from studies rated as 1++ or 1+

**C -** A body of evidence including studies rated as 2+ directly applicable to the target population and demonstrating overall consistency of results; or

Extrapolated evidence from studies rated as 2++

**D -** Evidence level 3 or 4

Extrapolated evidence from studies rated as 2+

## Appendix 2

Example of a theatre proforma for monitoring fluid management during operative hysteroscopy **Operative Hysteroscopy**
